# Diagnostic uncertainty of Takotusbo cardiomyopathy in a woman with cardiovascular risk factors hijacked at gunpoint: a case report

**DOI:** 10.1186/1757-1626-1-8

**Published:** 2008-05-23

**Authors:** Olagoke Akinwande, Yasmin Hamirani, Ashok Chopra

**Affiliations:** 1 Department of Medicine – St. Agnes Hospital, Baltimore MD, USA; 2 Department of Cardiology – St. Agnes Hospital, Baltimore MD, USA

## Abstract

**Background:**

Left ventricular apical ballooning (LVAB) is a type of cardiomyopathy precipitated by emotional or physiological stress, which results in transient left ventricular dysfunction and electrocardiographic (ECG) changes with relatively normal coronary arteries.

**Case presentation:**

We present a case describing the complexity of the diagnosis of LVAB in a patient with cardiovascular risk factors. We also review the literature from the initial discovery of this condition to the new developments.

**Conclusion:**

In patients with cardiovascular risk factors, diagnosis of LVAB is a challenge due to the similarities in symptoms with acute coronary syndrome. Such patients should be managed as an acute coronary syndrome until the definitive diagnosis is made. However, LVAB must still be maintained as a differential diagnosis in those patients to avoid unnecessary therapy.

## Background

Left ventricular apical ballooning (LVAB) mimics acute coronary syndrome in that it typically presents with chest pain or dyspnea with electrocardiographic (ECG) changes that are most commonly ST elevations or T-wave inversions. While coronary angiography reveals normal coronary arteries, left ventriculography shows transient wall motion abnormalities of the left ventricular apical and mid-ventricular aspects. Several physiological and emotional stressors have been implicated as precipitating factors in this condition. Through this case report, we will elucidate the fact that the presence of cardiovascular risk factors does not exclude a patient from having LVAB, but might in fact contribute to the pathomechanism. We also want to point towards the diagnostic challenge that occurs when non-significant coronary artery occlusion is documented in arteries not corresponding to the area of myocardial dysfunction. We present a patient who developed transient left ventricular apical ballooning after being hijacked at gunpoint.

## Case description

**Figure 1:**
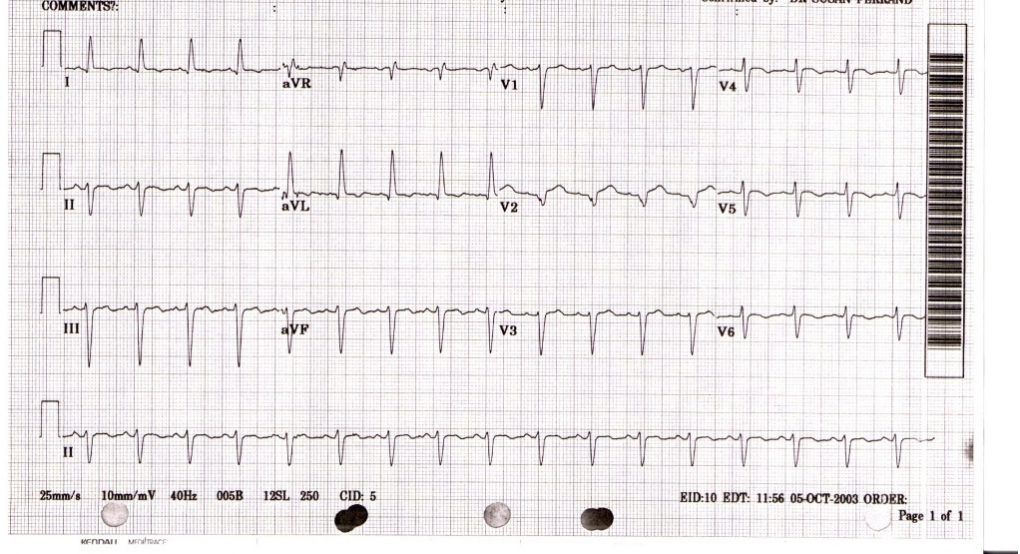
Initial EKG on arrival to the emergency department.

**Figure 2:**
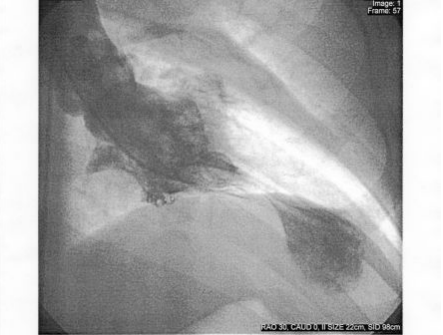
Left ventriculogram during systole displaying the characteristic apical ballooning with apical akinesis.

Our patient is a 77 year old Caucasian female with a past medical history of hypertension and peripheral vascular disease who presented with substernal chest discomfort. A few hours prior to admission the patient's car was hijacked at gunpoint. Shortly after being attacked, she suddenly developed a sudden squeezing chest pain accompanied with dyspnea and bilateral jaw pain. The pain was mild to moderate with no associated dizziness, diaphoresis, nausea or vomiting. On the way to the hospital, the patient received sublingual nitroglycerin with no relief. On arrival to the emergency department, her vital signs were within normal limits. On physical examination, no jugular venous distension or peripheral edema was noted. Her lung exam was normal. The cardiovascular exam revealed a tachycardia and normal S1 and S2 sounds. A systolic ejection murmur was appreciated at the apex with no radiation. No rubs or gallops were noted. ECG tracings revealed sinus tachycardia, non specific ST and T wave changes (see figure 1). Peak CK-MB and Troponin I levels were 12.3 ng/ml (0–4.9 ng/ml) and 3.8 ng/ml (0–0.2 ng/ml) respectively. Serum chemistries and pro BNP were normal. The patient was given aspirin, nitroglycerin, eptifibatide, beta blockers and intravenous heparin and was scheduled for cardiac catherization. Coronary angiography revealed 70% stenosis of the right coronary artery (RCA) immediately beyond the ostium which did not correlate with the area of myocardial compromise. The remainder of the vessel was normal giving rise to standard right ventricular and acute marginal branches. The left coronary artery (LCA) and its branches showed no significant stenosis. Left ventriculography showed left ventricular dilatation and dysfunction, with an ejection fraction of 30%. The distal halves of the anterior and inferior wall adjoining the apex were akinetic while the proximal segments displayed normal contraction (figure 2). The patient improved dramatically within hours of the event and was discharged in two days on aspirin, lisinopril, metoprolol and pravachol. Our patient improved dramatically the next day with normalization of her troponin I levels in 4 days. Two years later, the patient was re-evaluated with an echocardiogram which revealed normal left and right ventricular function with no wall motion abnormalities and an estimated ejection fraction of 60%.

## Discussion

Left ventricular apical ballooning (LVAB) is a type of cardiomyopathy characterized by transient wall motion abnormalities most commonly involving the apical and mid-ventricular portion of the ventricle. Patients typically present with findings similar to an acute coronary syndrome, but evaluation by cardiac catherization reveals non stenotic coronary arteries. Dote et al gave the moniker "Tako-tsubo" to this cardiomyopathy because the peculiar shape exhibited by the ventricle resembled a trap used in Japan to catch octopus [1]. One report estimated that about 2.2% of patients that present to the hospital with acute coronary syndrome (ACS) have LVAB. Most patients with LVAB are postmenopausal women, and the mean age is between 62 to 75 years [2,3]. The criteria for the diagnosis of LVAB is still ill-defined, however a systematic review by Bybee et al proposed the following criteria which includes: transient akinesis or dyskinesis of the apical and mid-ventricular segments in association with regional wall motion abnormalities that extend beyond the distribution of a single pericardial vessel, absence on angiography of obstructive coronary artery disease or evidence of acute plaque rupture, new ST segment elevation or T wave inversion on the ECG, and absence of recent significant head trauma, intracranial bleeding, pheochromocytoma, myocarditis, or hypertrophic cardiomyopathy [3].

The etiology of LVAB is not well understood. The discovery of "Takotsubo like" phenomena in patients with medical conditions that caused increase in plasma catecholamines such as pheochromocytoma and significant head trauma, suggested the role of elevated plasma catecholamines in the pathogenesis of LVAB [4,5]. At the molecular level, Ueyama et al. experimented with an animal model using rats and was able to reproduce the ECG and left ventriculogram findings. They were able to show transient up-regulation of immediate early genes in the perfused heart attributed to activation of alpha- and beta- adrenoceptors [6]. Despite this laudable research milestone, the precise mechanism as to exactly how elevated plasma catecholamines leads to LVAB is yet to be determined.

Variants of LVAB syndrome with predominant mid-ventricular or basal involvement have been reported [7]. Perhaps the variations in regional wall motion observed in those variants and other entities involving excess catecholamines (pheochromocytoma and neurocardiogenic injury) relate more to differences in the anatomic location of cardiac adrenergic receptors, the degree of excess sympathetic activity involved or the differing susceptibilities to such sympathetic stimulation from individual to individual. Hypertrophic cardiomyopathy with apical aneurysm is another condition that could mimic LVAB anatomically [8].

The role of ischemia in the pathogenesis of LVAB was elucidated by nuclear studies which compared LVAB to acute myocardial infarction (AMI). Using 99mTc-tetrofosmin, 123I-BMIPP, and 123I-MIBG as markers for myocardial perfusion, myocardial fatty acid metabolism and myocardial sympathetic nerve function respectively, the study which measured myocardial perfusion scores displayed striking similarities in between the LVAB and the AMI groups in the acute phase (2–14 days). Complete normalization of myocardial perfusion scores was achieved after 3 months in the group with LVAB, but remained significantly abnormal in the group with AMI. These results suggested that LVAB was due to a stunned myocardium with a plausible role of ischemia as a causative mechanism [9]. Oddly, patients with LVAB are typically devoid of significant coronary artery stenosis on angiography. The theories explaining myocardial ischemia with a negative angiogram include epicardial coronary arterial spasms, microvascular spasms, impaired fatty acid metabolism, myocarditis, transient obstruction to LV outflow, catecholamine mediated myocardial dysfunction and acute coronary syndrome with spontaneous coronary recanalization [10,11]. The theory of spontaneous recanalization was suggested by Ibanez and colleagues in a paper in which they used intravascular ultrasound to examine coronary patency in five patients with LVAB. In all the patients they examined, they found evidence of disrupted plaques in the left anterior descending artery (LAD) in patients with LVAB which suggested the etiology of LVAB was probably due to ACS with early recanalization leading to myocardial stunning. Elevated catecholamines increased the propensity for plaque rupture and subsequent thrombus formation. This study was instrumental in illustrating that a negative angiogram does not rule out coronary artery disease in patients with LVAB [12].

Patients with LVAB commonly present with chest pain (67%) and/or dyspnea. ECG manifestations include ST-segment elevation or depression most commonly in the precordial leads (V2 to V6), T-wave inversions (localized or diffuse), pathologic Q waves and prolonged Q-T intervals. Cardiac enzymes and brain type natriuretic peptide (BNP) are elevated in most patients with this condition. Echocardiography typically shows wall motion abnormalities including apical akinesis/dyskinesis, hyperkinesis of the basal segment (occasionally) and reduced ejection fractions. Coronary angiography shows coronary arteries devoid of any significant occlusive disease and left ventriculography reveals characteristic apical, midventricular, and sometimes basal wall motion abnormalities [13-16]. Cardiac magnetic resonance imaging usually shows lack of delayed gadolinium hyper-enhancement which indicates viability of the involved myocardium.

The prognosis of patients with LVAB is favorable. The mortality rate in the largest study we found (n = 88) was 1% [11]. Patients usually enjoy complete resolution of the cardiac structural abnormalities, but in some cases, sequelae such as mitral regurgitation, ventricular arrhythmias, cardiogenic shock and very rarely death may occur. Treatment for patients with LVAB is supportive with medications like diuretics, ACE inhibitors and beta-blockers depending on the symptomatology [3]. Patients who are in shock should undergo urgent echocardiography to determine if LVOT obstruction is present. If present, inotropes should be avoided as it might worsen the degree of obstruction. In these patients, beta blockers and fluid resuscitation (in the absence of pulmonary congestion) might be indicated. If obstruction is absent the patient might benefit from inotropes or intra-aortic balloon counterpulsation (IABP) [3,16,17]. In the event of life threatening arrhythmias, the implantation of a cardioverter-defibrillator has to be considered.

The timing of the emergence of our patient's symptoms coinciding with the emotion stressor caused by the car hijacking makes LVAB the most likely diagnosis. The presentation was typical; chest pain, dyspnea, ST and T wave anomalies, elevated cardiac enzymes, non-stenotic corresponding coronary arteries and a characteristic ventriculogram. A limitation to our case that made the diagnosis tougher is the fact that our patient was non compliant with immediate follow up after discharge, so we were unable to document wall motion abnormalities in the acute and subacute phases. However, in the chronic phase, wall motion abnormalities and ejection fraction by echocardiogram did normalize.

Another factor that adds to a more burdensome diagnosis is that the patient had significant stenosis of the RCA. But we argue that even though there was significant stenosis seen in the right coronary artery, the region of blood supply did not correspond to the zone of left ventricular dysfunction. The left coronary artery which supplied the dysfunctional myocardium showed no stenosis, but it is noteworthy that coronary angiography is not a sensitive enough modality to determine if occlusion with recanalization took place. Hence, even though our patient had a clear emotional stressor, we cannot completely rule out ACS with recanalization via angiography; perhaps this diagnosis might have been aided by the use of intravascular ultrasound. Nevertheless, we can speculate based on the paper by Ibanez et al that the emotional stressor might act in concert with a disrupted plaque to explain the pathomechanism of our patient's condition. This case illustrates the complexity of the diagnosis of this ill-defined condition in this subset of patients.

## Conclusion

Patients that present with symptoms suggestive of ACS should be treated as such, due to the overlap in the management of both conditions. In patients with cardiovascular risk factors, ACS is more likely, however LVAB should also be considered in the differential. Good knowledge of this condition gives good prognostic insight for patient education and appropriate management tailoring.

## Competing interests

The authors declare that they have no competing interests.

## Authors' contributions

OA conceived the study, substantially involved in the acquisition of data, compilation of relevant literature, and drafted the preliminary and final manuscript.

YH was involved intellectually the revision, formatting and proofreading the manuscript.

AC cared for the patient, and was involved in the provisional and final diagnosis. He also reviewed the manuscript.

All authors have read and approved the final version of the manuscript

## Consent

Written informed patient consent was obtained from the patient for publication of this case report. A copy of this consent is available for the Editor-in-Chief upon request.
